# Implications of Partial Conjugation of Whey Protein Isolate to Durian Seed Gum through Maillard Reactions: Foaming Properties, Water Holding Capacity and Interfacial Activity

**DOI:** 10.3390/molecules181215110

**Published:** 2013-12-06

**Authors:** Bahareh Tabatabaee Amid, Hamed Mirhosseini, Hashem Poorazarang, Seyed Ali Mortazavi

**Affiliations:** 1 Department of Food Technology, Technology, University Putra Malaysia, UPM Serdang 43400, Selangor, Malaysia; E-Mail: bahareh.ta@gmail.com; 2 Department of Food Science and Technology, Ferdowsi University of Mashhad (FUM), P.O. Box, Mashhad 91775-1163, Iran; E-Mails: hashempoorazarang1996@gmail.com (H.P.); mortazavi1994@gmail.com (S.A.M.)

**Keywords:** conjugation process, Maillard reaction, durian seed gum, whey protein isolate, particle uniformity, specific surface area

## Abstract

This paper deals with the conjugation of durian seed gum (DSG) with whey protein isolate (WPI) through Maillard reactions. Subsequently, the functional properties of durian seed gum in the non-conjugated (control sample) and conjugated forms were compared with several commercial gums (*i.e.*, Arabic gum, sodium alginate, kappa carrageenan, guar gum, and pectin). The current study revealed that the conjugation of durian seed gum with whey protein isolate significantly (*p* < 0.05) improved its foaming properties. In this study, the conjugated durian seed gum produced the most stable foam among all samples. On the other hand, the emulsion stabilized with the conjugated durian seed gum also showed more uniform particles with a larger specific surface area than the emulsion containing the non-conjugated durian seed gum. The conjugated durian seed gum showed significant different foaming properties, specific surface area, particle uniformity and water holding capacity (WHC) as compared to the target polysaccharide gums. The conjugated durian seed gum showed more similar functional properties to Arabic gum rather than other studied gums.

## 1. Introduction

Proteins and polysaccharides are utilized in the formulation of the food products as a foam stabilizer, texture, and rheology modifier [1,2]. Some of the proteins have both hydrophobic and hydrophilic regions responsible for reducing the surface tension via the interaction at the emulsion interface [3]. This interface activity depends on several factors such as the protein’s solubility and the attractive-repulsive balance between the aqueous phase environment and proteins [4]. The emulsifying activity of the protein against aggregation and coalescence is mainly due to the steric and electrostatic interactions [5]. Proteins also have a good ability to produce stable foams. In this regard, the possible conformation rearrangements along with the hydrophobicity characteristic of the protein could be responsible for its foaming properties [6,7]. Proteins can provide a film which surround the gas bubbles and keep them in food products [7]. The stability of protein’s foam depends on many factors such as drainage (from lamellae and plateau boarders), and bubble coalescence [8]. As stated by Shepherd *et al*. [3], the emulsifying activity of some of the proteins (such as casein, soy protein and commercial whey protein) at the isoelectric points becomes lower than their original forms. This may cause a limitation on their usage in the acidic foods [9].

On the other hand, some of the polysaccharide gums (such as Arabic gum) have both foaming and interface activity. They can migrate slowly to air-water and oil-water interfaces, and make stable foams and emulsions [10]. However, most of the polysaccharides do not have an ability to interact with the oil phase in emulsions and act as an emulsifier because of the lack of hydrophobic moieties in their molecular structure [3]. In some products, this problem has been solved through the simple mixing, coacervation and/or conjugation of the polysaccharide with an appropriate protein. It is well known that the functional properties of polysaccharide and protein could be further enhanced by the covalent linkage of the target protein and polysaccharide [11]. In most cases, the conjugation of protein to the polysaccharide has been carried out by dry-heating techniques [12]. The Maillard reaction can convert unusable food materials to valued food ingredients [13]. Maillard-type reactions between the amino groups and carbonyl groups are responsible for the graft reaction between the protein and polysaccharide [14,15]. This phenomenon can be explained by the linkage of the terminal and interstitial amines of the protein to the reducing sugar through the Amadori rearrangement [3]. As reported by previous researchers [12,13,16,17,18], protein-polysaccharide conjugates showed better functional properties than the polysaccharides alone. Yadav and his colleagues [16] showed that corn fiber gum conjugated with proteins through Maillard reactions had better emulsifying ability than corn fiber gum or protein at high salt concentration and under acidic conditions. Mu *et al*. [12] reported that soy protein-acacia gum conjugate showed better solubility than the soy protein isolate and its mixture with acacia gum at the same pH value. They stated that soy protein-acacia gum conjugate provided more stable emulsions with the smaller droplet sizes than soy protein and acacia gum alone [12].

Durian seed gum (DSG) is a heteropolysaccharide-protein biopolymer with a relatively large molecular weight ranging from 1.08 × 10^5^ to 1.44 × 10^5^ (g/mol) [19]. Galactose (48.6%–59.9%), glucose (37.1%–45.1%), arabinose (0.58%–3.41%), and xylose (0.3%–3.21%) are the main mono-saccharides in the molecular structure of durian seed gum. In addition, the crude durian seed gum contains trace amounts of protein and lipid fractions [19]. Our previous study revealed that the predominant fatty acids of the lipid fraction in durian seed gum were palmitic acid (C16:0), palmitoleic acid (C16:1), stearic acid (C18:0), oleic acid (C18:1), linoleic acid (C18:2), and linolenic acid (C18:2). In addition, the main amino acid components in the protein fraction of durian seed gum were leucine (30.9%–37.3%), lysine (6.04%–8.36%), aspartic acid (6.10%–7.19%), glycine (6.07%–7.42%), alanine (5.24%–6.14%), glutamic acid (5.57%–7.09%), valine (4.5%–5.50%), proline (3.87%–4.81%) and serine (4.39%–5.18%) [19]. It has been proven that durian seed gum has the ability to stabilize oil in water emulsions [20]. The foaming ability and emulsifying properties of durian seed gum may not be comparable with commercial gums. It was hypothesized that the conjugation of durian seed gum with whey protein isolate (WPI) through the Maillard reaction might improve its functional properties. The present study focused on the preparation of durian seed gum (DSG) linked to whey protein isolate (WPI) through the conjugation process. In this study, the functional properties of durian seed gum in the single and conjugated forms with whey protein isolate (WPI) were compared with several commercial gums to investigate the possible similarity and/or differences between durian seed gum in the single and conjugated forms with these commercial gums. Target polysaccharide gums were Arabic gum, sodium alginate, kappa carrageenan, guar gum, and pectin. The current research mainly investigated the foam properties, water holding capacity, and particle size and uniformity of durian seed gum as compared to the commercial polysaccharide gums. To the best of the knowledge, there is no similar study reported the foam properties and other functional properties of durian seed gum in the single and conjugated form in an aqueous system.

## 2. Results and Discussion

### 2.1. Water-Holding Capacity (WHC) of Conjugated Durian Seed Gum and Various Gums

**Figure 1:**
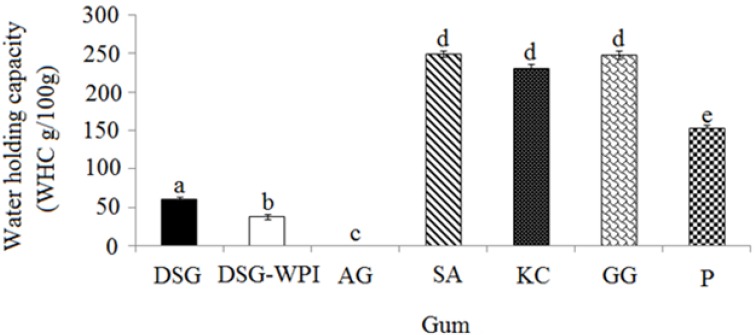
Water holding capacity (WHC) of the non-conjugated durian seed gum (DSG) and conjugated durian seed gum (DSG-WPI) as compared to the target commercial gums (*i.e.*, Arabic gum (AG), sodium alginate (SA), kappa carrageenan (KC), guar gum (GG), and pectin (P); **a**–**e**: significant different at *p* < 0.05.

Most of the plant polysaccharide gums are water-soluble polymers. They can absorb water and swell up to form a gel or provide highly viscous solutions when added to water [21]. This is one of the most critical characteristics of polysaccharide gums, which significantly affects their functional applications. The amount of water held by one gram of gum is known as the water holding capacity (WHC). The binding strength of different polysaccharide gums mainly depends on their molecular structure [22]. Therefore, the changes in the molecular structure of a polysaccharide gum may significantly alter its water holding capacity. The results showed that the conjugation of whey protein isolate to the molecular structure of durian seed gum significantly (*p* < 0.05) reduced its ability to hold water and the conjugated durian seed gum had much lower water holding capacity than sodium alginate, kappa carrageenan, guar gum, and pectin (Figure 1). The water holding capacity of the conjugated hybrid polymer depends on the conjugation condition, degree of graft reaction between the protein and polysaccharide and the surface hydrophobicity of the hybrid polymer. If the conjugation process decreases the surface hydrophobicity of the conjugated hybrid polymer, it will provide stronger water holding ability than is its original form. Conversely, if the conjugation process increases the surface hydrophobicity, the conjugated hybrid polymer will induce a weaker water holding ability than in its original form. On the other hand, if the degree of graft reaction increases by prolonging the conjugation process, stronger linkages will be formed between the polysaccharide and protein. The type and content the linkage between the polysaccharide and protein significantly affect the water holding ability of the conjugated hybrid polymer. The results showed that there was a significant (*p* < 0.05) difference among the target polysaccharide gums in term of the water holding capacity. This difference might be due to: (i) the type and content of functional hydrophilic groups present in the carbohydrate portion of the gum; (ii) the number of hydration positions; and (iii) the interaction between the gum and water molecules [23]. This ability might be also influenced by the presence of a small portion of protein in the molecular structure of the gum and functional groups involved in binding water molecules [24].

In the current study, Arabic gum showed the weakest water holding capacity. In fact, Arabic gum holds an almost negligible amount of water and consequently it does not have the ability to produce a gel (Figure 1). The low water holding capacity of Arabic gum might be due to its high solubility even at high concentration. The solubility of Arabic gum may be due to the presence of many hydroxyl groups and the nature of the molecular structure of its monosaccharides [25]. Conversely, guar gum and sodium alginate had the greatest capacity to hold water. When the polysaccharide gum holds water, the trapped water decreases the entropy; therefore the water has to form bonds with a more negative enthalpy. This process results in stronger linear hydrogen bonds, thereby causing larger structures with a lower density than the original form. The same effect is caused by capillary action and reduced osmotic pressure. This will result in “stretching” the water molecules into the tetrahedral structure of the polymer [26].

### 2.2. Foam Capacity and Stability of Conjugated Durian Seed Gum and Various Gums

**Figure 2:**
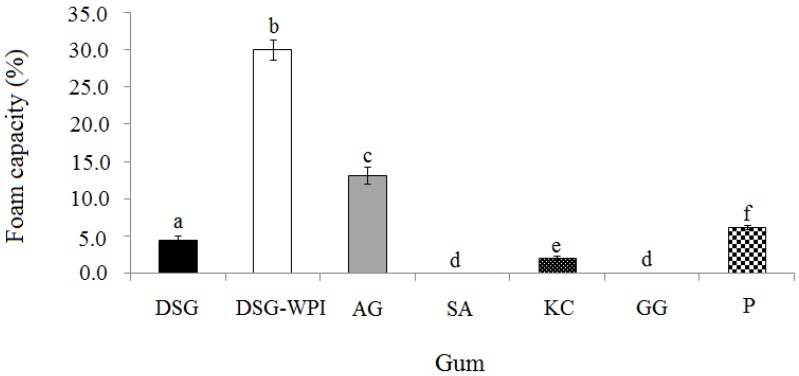
Foaming capacity of the non-conjugated durian seed gum (DSG) and conjugated durian seed gum (DSG-WPI) as compared to other commercial gums (*i.e.*, Arabic gum (AG), sodium alginate (SA), kappa carrageenan (KC), guar gum (GG), and pectin (P); **a**–**f**: significant different at *p* < 0.05.

**Figure 3:**
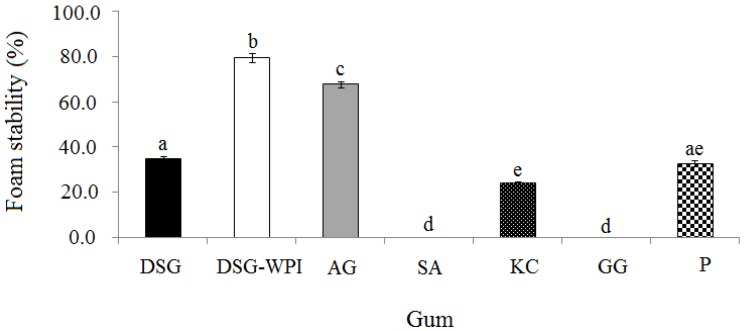
Foaming stability of the non-conjugated durian seed gum (DSG) and conjugated durian seed gum (DSG-WPI) as compared to other commercial gums (*i.e.*, Arabic gum (AG), sodium alginate (SA), kappa carrageenan (KC), guar gum (GG), and pectin (P); **a**–**e**: significant different at *p* < 0.05.

The foam capacity and stability induced by durian seed gum, the conjugated durian seed gum and other commercial gums are displayed in Figure 2 and Figure 3, respectively. The results showed that the target polysaccharide gums exhibited significantly different (*p* < 0.05) foam abilities (Figure 2). This might be due to their significantly different chemical structures and molecular weights. The current study showed that sodium alginate and guar gum, which showed the strongest water holding capacity, exhibited the weakest foaming ability among all target polysaccharide gums (Figure 2). The conjugated durian seed gum had much higher foam ability than its original form (Figure 2). This confirms the significant positive effect of conjugation process with whey protein isolate on the foam ability of durian seed gum. Walsh *et al*. [27] also showed that protein could play a significant role in the formation and stabilization of a dispersed gas phase. As a result of the conjugation process, protein-polysaccharide hybrid polymers have more ability to form a viscoelastic layer at the bubble surface and prevent the rupture of the film and Ostwald ripening [28]. The ability of the protein to make foam depends on its characteristics, structure and composition (*i.e.*, the hydrophobicity, compact globular structure rearrangement, polar and non-polar regions). For instance, the presence of hydrophilic groups in the molecular structure of protein leads to the attraction of the water phase; whereas the hydrophobic groups present in the protein are arranged towards the air phase [29]. The protein characteristics also play a significant role in its adsorption rate at the air-water interface and its ability to form a coherent elastic adsorbed layer [6]. The present study showed that Arabic gum also had the significant (*p* < 0.05) strong foaming ability. It had a lower foaming capacity than the conjugated durian seed gum, but higher than the non-conjugated durian seed gum and other target polysaccharide gums. The foaming ability of Arabic gum may be explained by the presence of arabinogalactan-protein (AGP) fraction in the molecular structure of Arabic gum. In fact, the presence of these surface active groups in the molecular structure of the gum leads to reduce the surface tension and give the ability to form a film at the surface of the gas bubbles [30,31].

The stability of foam is one of the main concerns in the formation of foams. Figure 3 represents the differences of stability of the foams formed by the conjugated and non-conjugated durian seed gum as compared to different polysaccharide gums. The results revealed that the conjugated durian seed gum and Arabic gum provided more stable foams than the non-conjugated durian seed gum and other target polysaccharide gums.

As shown in Figure 2 and Figure 3, sodium alginate and guar gum did not provide stable foam, most probably due to the highly viscous solution they form. Prabhanfan *et al*. [32] also reported that guar gum could produce a small amount of foam at a certain concentration. The results showed that pectin and the non-conjugated durian seed gum provided poorly stable foam as compared to Arabic gum and the conjugated durian seed gum. It might be due to the amphiphilic structure of Arabic gum and the conjugated durian seed gum. This could be explained by the presence of protein which reduces the surface tension resulting in the formation of a stable foam [33]. Most of the polysaccharides do not have the ability to absorb at the interface due to their hydrophilicity, whereas their specific properties such as thickening and gelling are responsible for making stable foama when the protein is added to an aqueous solution [34,35]. The main factors affecting the foam stability are the adsorption rate, surface tension and length of the surfactant chain. As stated by Damodaran [9], the protein responsible for forming the stable foam must have the ability to absorb at the air-water interface quickly. It also must have the ability to denature promptly at the interface to keep the balance between the degrees of hydrophobicity and hydrophilicity. From another point of view, drainage, and coalescence also influence the stability of foams [31]. There are different mechanisms describing the foam rapture. The first mechanism is the air diffusion from the interior region resulting in the bubble size reduction and consequently reducing the foam stability. The second reason for foam rapture is to form some holes between two bubbles through the rupture of lamellae. The third mechanism responsible for the rapture of foam is the drainage of the water surrounding the air bubbles [29].

In this study, the formation of air bubbles and foaming capacity decreased with increasing viscosity of the continuous phase during stirring. Prabhanfan *et al*. [32] also reported the similar observation. During the mechanical stirring, air comes into the solution to form bubbles. The presence of hydrophobic regions in the protein polymer structure facilitates its adsorption at the interface, resulting in partial unfolding (or surface denaturation). The change in the molecular configuration of the protein reduces the solubility and results in the precipitation of proteins at the liquid-air interface. The presence of the unfolded protein fraction at the liquid-air interface leads to a reduction of the surface tension and facilitates the formation of more stable air bubbles. In fact, the presence of partially unfolded protein molecules at the liquid-air interface results in the formation of a stable film around the air bubbles [29], so the unfolded protein fraction should absorb at the interface and form a strong cohesive, viscoelastic film. This viscoelastic film can resist the thermal and mechanical rapture of the foam.

### 2.3. Droplet Size Uniformity of Differently Formulated Emulsions

**Figure 4:**
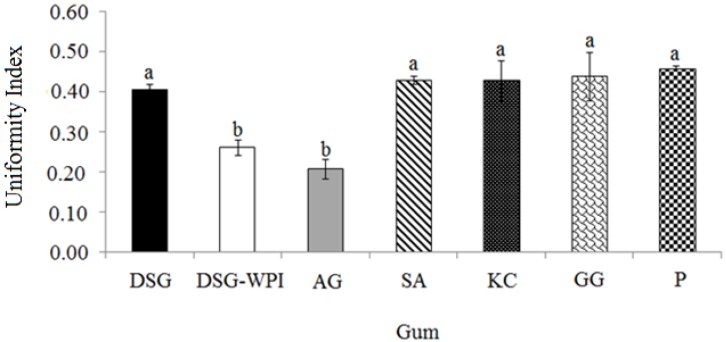
Particle uniformity of the non-conjugated durian seed gum (DSG) and conjugated durian seed gum (DSG-WPI) as compared to other commercial gums (*i.e.*, Arabic gum (AG), sodium alginate (SA), kappa carrageenan (KC), guar gum (GG), and pectin (P); **a**,**b**: significant different at *p* < 0.05.

The stability and viscosity, texture, and chemical activity of an emulsion significantly depend on its droplet size and distribution [36,37,38,39]. The use of an appropriate emulsifier depends on its effects on the droplet size and distribution of the emulsion [40]. An emulsifier providing smaller emulsion droplets with more droplet uniformity can cause more stable emulsions. Figure 4 compares the droplet uniformity of differently formulated emulsions stabilized with the non-conjugated and conjugated durian seed gum, Arabic gum, sodium alginate, kappa carrageenan, guar gum and pectin. In this study, a higher uniformity index from the particle size analyzer refers to lower droplet uniformity (Figure 4). The results showed that the droplet uniformity of oil in water (O/W) emulsions containing durian seed gum and Arabic gum were significantly (*p* < 0.05) different from the other commercial gums (Figure 4). This phenomenon could be due to the diversity of the target commercial gums applied for stabilization of O/W emulsions. The considerable effect of polysaccharide gums on the emulsion stability is mainly due to their significant effects on the droplet size and viscosity of the emulsion. In fact, they prevent oil droplet collision by enhancing the viscosity of the aqueous phase [5,41].

There was no significant (*p* > 0.05) different among the droplet uniformity of emulsions containing sodium alginate, kappa carrageenan, guar gum, pectin and non-conjugated durian seed gum. In this study, the emulsions stabilized with pectin and guar gum had the highest uniformity index (or the least droplet uniformity) among all prepared emulsions. This indicated that they exhibited very low emulsifying activity. Al-Hakkak and Al-Hakkak [42] also reported that pectin showed weak emulsifying activity. The researchers reported that the emulsion stabilized with pectin was stable within 10 days from the production date. However, the emulsifying activity of pectin depends on many factors such as the content of acetyl groups, molecular weight and the presence of calcium ion [43,44]. The results revealed that the emulsions stabilized with the conjugated durian seed gum and Arabic gum had the most uniform droplets among all prepared emulsions. This might be due to the presence of the protein fraction in the molecular structure of the conjugated durian seed gum and Arabic gum. Mu and his colleagues [12] also reported the similar observation for acacia gum conjugated with soy protein isolate. They illustrated that the emulsion containing Arabic gum (AG) conjugated with soy protein isolate (SPI) had the smaller droplet size distribution than the emulsion stabilized with the non-reacted SPI and Arabic gum. Mu *et al*. [12] also demonstrated that the mixture of soy protein isolate and Arabic gum caused more uniform emulsion droplets than soy protein isolate and Arabic gum alone.

### 2.4. Specific Surface Area of Differently Formulated Emulsions

**Figure 5:**
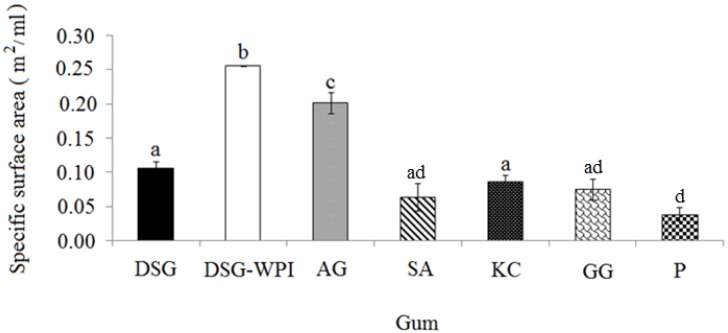
Specific surface area of the non-conjugated durian seed gum (DSG) and conjugated durian seed gum (DSG-WPI) as compared to other commercial gums (*i.e.*, Arabic gum (AG), sodium alginate (SA), kappa carrageenan (KC), guar gum (GG), and pectin (P); **a**–**d**: significant different at *p* < 0.05.

The specific surface area (the surface area of all emulsion droplets per unit volume of emulsion) is an indicator to determine the emulsifying activity of an emulsifier. In order to have a stable emulsion, the minimum emulsion droplet size and maximum surface area per unit volume of oil are required [6,45]. In the present study, the specific surface area of durian seed gum and target polysaccharide gums was compared. Figure 5 shows the specific surface area of differently formulated emulsions containing different polysaccharide gums. The results indicated that the polysaccharide gums (*i.e.*, sodium alginate, kappa carrageenan and guar gum) with weaker foaming capacity exhibited lower emulsion stability too (Figure 1 and Figure 5). Conversely, the polysaccharide gums with the stronger foaming ability (*i.e.*, the conjugated durian seed gum and Arabic gum) showed higher emulsifying activity. It should be noted that the molecular weight and hydrophobic to hydrophilic ratio of the polysaccharide gum significantly affect its migration and absorption at the oil-water interface. This might be responsible for significant differences among the differently formulated emulsions in term of the specific surface area. On the other hand, the emulsifying activity of polysaccharide gums may be due to the presence of the proteinaceous moiety in their molecular structure [46]. In our previous study [47], sodium dodecyl sulfate (SDS)-polyacrylamide gel electrophoresis (SDS-PAGE) was applied to verify the linkage of protein fraction to DSG through the conjugation process. The results revealed that the covalent linkage between durian seed gum and whey protein isolate through Maillard reactions significantly affected the molecular structure and functional properties of both polymers in water in oil in water (W/O/W) emulsion [47]. The conjugated durian seed gum had darker colour than the non-conjugated gum. In fact, the reaction between the amino groups from amino acids and a glycosidic hydroxyl group of the reducing monosaccharide results in the formation of brown nitrogenous polymers such as melanoidins [47].

The present study showed that the graft reaction between whey protein isolate and durian seed gum resulted in the new hybrid polymer with much stronger interface activity than durian seed gum alone. It was hypothesized that the covalent linkage of whey protein isolate to the structure of durian seed gum resulted in the formation of the hybrid polymer with more hydrophobic-hydrophilic structure than its original form. This might be responsible for the larger specific surface area formed by the hybrid polymer. As shown in Figure 5, the conjugated durian seed gum produced the most stable emulsions among all target gums. Dickinson [45] also revealed that the protein-polysaccharide conjugate had more surface activity than the polysaccharide itself. In fact, the polysaccharide-protein conjugate can form a bulkier polymeric layer on the surface of oil droplets in the emulsion [5,47]. The conjugated polymer can provide stronger steric and electrostatic repulsive forces among oil droplets, thus inhibiting them from the droplet collision and inducing more efficient emulsifying activity than the polysaccharide or protein alone.

The results showed that the emulsions stabilized with sodium alginate, kappa carrageenan, pectin and guar gum did not have significantly different specific surface areas. They showed relatively weak emulsifying activity. As also stated by Dickinson [48], some of the polysaccharides are not suitable emulsifiers due to their hydrophilic character. On the other hand, the non-conjugated durian seed gum provided a relatively better emulsifying activity than sodium alginate, kappa carrageenan, guar gum, and pectin. The emulsifying activity of the non-conjugated durian seed gum could be due to the presence of a low content of protein (<4%) in its molecular structure [19,49]. Dickinson [48] also illustrated that the attachment of a small fraction of surface active protein to the gum could be responsible for its emulsifying activity. In fact, the absorption of a thin layer of surface-active protein at the oil-water interface could stabilize the oil droplets. On the other hand, this attachment most probably results in the large size polymer with high hydrophilicity, which generates a long range steric repulsion between emulsion droplet surfaces [45]. Our previous study [47] also showed that the conjugated durian seed gum produced higher viscosity than the non-conjugated gum. This observation also confirmed the formation of the covalent linkages between durian seed gum and whey protein isolate, resulting in the new hybrid polymer with the larger molecular weight than its original form.

## 3. Experimental

### 3.1. Chemicals and Materials

Fully ripe durian (*D. zibethinus*) fruits and soybean oil were purchased from a local market and supermarket (Selongor, Malaysia). In the current study, gum Arabic, kappa-carrageenan, sodium alginate, pectin and guar gum were supplied by Sigma-Aldrich (St. Louis, MO, USA). Isopropanol, ethanol (95% and 99.9%), acetic acid, acetone, saturated barium hydroxide, sodium benzoate, potassium sorbate and citric acid were purchased from Fisher Scientific (Pittsburgh, PA, USA). Whey protein isolate was supplied by Glanbia Nutritionals (Monreo, WI, USA).

### 3.2. Isolation and Purification of Durian Seed Gum

After de-husking the ripped fruits, the seeds were removed, cleaned and rinsed with sterile water. The rinsed seeds were subjected to circulating air at room temperature to decrease the surface moisture content and avoid germination during storage. The air dried seeds were packed in the tight plastic bags and stored in a dry and cool place (10 ± 2 °C). Durian seed gum was isolated from the defatted-decolorized durian seed flour according to the method reported earlier [50]. Briefly, the durian seed flour was soaked in 1% aqueous acetic acid at room temperature (25 ± 1 °C) for 1.5 h. The sample was filtered through nylon cloth filter, and the filtrate was precipitated with 95% ethanol. Then, the precipitate was collected and washed with absolute ethanol (99.0%) to obtain a very light brown amorphous crude gum [50]. The crude durian seed gum was dried by a freeze dryer at −40 °C. The lyophilized crude durian seed gum was milled and sieved using a mesh 18 sifter. The purification of durian seed gum was performed using saturated barium hydroxide solution as reported in the previous research [51]. The solution (2.5%, w/v) containing the crude durian seed gum was stirred under the specified conditions (60 °C for 12 h). Then, the gum solution was precipitated with a saturated barium hydroxide solution, and centrifuged in an Avanti J-25 centrifuge (Beckman, Fullerton, CA, USA) for 15 min at 15,180 g. In the next step, the precipitate was stirred with 1 M acetic acid for 8 h, and centrifuged for 15 min at 15,180 g. The supernatant was precipitated and washed with 95% ethanol. Finally, the sample was freeze-dried according to the method described earlier [51].

### 3.3. Coupling of Durian Seed Gum with Whey Protein Isolate

Dry-heating reactions between durian seed gum and whey protein isolate were conducted at the ratio of 3:1 (W/W). Then, the sample was dried in a freeze dryer, and ground to make the powder. The conjugation reaction was performed by incubating the lyophilized sample in the oven at 60 °C with relative humidity of 80% for 48 h. After incubating samples under the specific conditions, the sample color turned light brown. This might be due to a moderate degree of non-enzymatic browning reaction. Finally, the conjugated polymer was placed in the freezer until use [17].

### 3.4. Preparation of Water in Oil (W/O) Emulsion

In this study, different oil-in-water emulsions (O/W) were prepared by using the non-conjugated and conjugated durian seed gum and several commercial gums (*i.e.*, Arabic gum, sodium alginate, kappa-carrageenan, guar gum and pectin) as an emulsifier. The other components were preservatives (*i.e.*, sodium benzoate, 0.1%; potassium sorbate, 0.1% w/w), citric acid (0.5% w/w), corn oil (5% w/w) and deionized water. In the first step, the continuous phase was prepared by dispersing sodium benzoate, potassium sorbate and citric acid in deionized water and subsequently blending with a high-speed blender (32BL80, Waring, Torrington, CT, USA). While mixing, a similar content (5% w/w) of each gum was gradually added to the blender and constantly mixed for 3 min. The mixture was kept overnight at room temperature to facilitate the hydration of the continuous phase. Then, corn oil was gradually added into the hydrated continues phase and mixed for 5 min to make a coarse emulsion [49]. Finally, the coarse emulsion was passed through a high pressure homogenizer (APV, Crawley, UK) for three passes (30, 28 and 25 MPa) to prepare a fine emulsion [52].

### 3.5. Analytical Tests

#### 3.5.1. Water-Holding Capacity (WHC)

In the present study, water holding capacity (WHC) of durian seed gum, the conjugated durian seed gum and other commercial gums was determined according to the method described by previous researchers [53]. Briefly, each gum (1 g) was suspended in distilled water (10 mL) and mixed for 2 min at the ambient temperature. Then, the samples were centrifuged with a refrigerated centrifuge (Sigma 3-18, Sartorius, Gottingen, Germany) at 3,000 g for 30 min. Finally, the free water was removed and the water content (g) absorbed by 100 g of the gum was considered as the water holding capacity [53].

#### 3.5.2. Foaming Properties

Foaming capacity and stability of durian seed gum, the conjugated durian seed gum and other commercial gums were measured according to the method described by previous researchers [54,55] with some minor modification. In this experiment, each gum (2 g) was dissolved in distilled water to provide 2% (w/w) solutions. Subsequently, the pH was adjusted to 6 and then 60 mL solutions were whipped at 15,000 rpm at the ambient temperature for 2 min with a high speed mixture (APV). This inhibits the denaturation and precipitation of the protein fraction during the experiment. Foaming capacity and stability were measured immediately after whipping at the ambient temperature. The experiment was carried out in triplicate for each sample. Foaming capacity was determined based on the following equation [55]:

Foam capacity (%) = [X − X_1_/X_1_] × 100

X = Volume after whipping(1)

X_1_ = Volume prior to whipping

Foaming stability was measured 30 min after preparing the foam based on the following equation [55]:

Foam stability = [V/V_1_] × 100

V = Foam valume after 30 min(2)

V_1_ = Total foam volume

#### 3.5.3. Specific Surface Area

In this study, the specific surface area (SSA) was measured at ambient temperature by using a particle size analyzer equipped with a Hydro 2000S accessory (Malvern Mastersizer 2000, Malvern Instrument Ltd., Worcestershire, UK). In order to prevent multiple scattering effects, all prepared emulsions were diluted (1:100, w/w) with the deionized water before measuring the specific surface area. The specific surface areas of fresh emulsions were carried out in triplicate for each sample [49].

#### 3.5.4. Analysis of Particle Uniformity

The particle uniformity was determined according to the method described by previous researchers [56,57]. It was measured by a Malvern particle size analyzer equipped with a Hydro 2000S accessory (Malvern Mastersizer 2000) at ambient temperature. The uniformity measurement was measured in duplicate for each sample. The measurement was carried out immediately after preparing the emulsion.

### 3.6. Experimental Analysis

In the present study, functional properties of durian seed gum in the single and conjugated forms were compared with several commercial gums (*i.e.*, Arabic gum, sodium alginate, kappa carrageenan, guar gum, and pectin). A completely randomized method was used to investigate the functional properties of durian seed gum in the single and conjugated forms as compared to several commercial gums (*i.e.*, Arabic gum, sodium alginate, kappa carrageenan, guar gum, and pectin). One way analysis (ANOVA) was used to determine the significant (*p* < 0.05) difference among polysaccharide gums in the aqueous system and emulsion [58]. Minitab version 15 (Minitab Inc., State College, PA, USA) was used to create the experimental design and run Fisher multiple comparison tests.

## 4. Conclusions

The current research studied the emulsifying activity, foaming capacity and water holding capacity of durian seed gum before and after conjugation. In addition, the functional properties of durian seed gum in the single and conjugated forms were compared with several polysaccharide gums. The present study revealed that the graft reaction between durian seed gum and whey protein isolate resulted in a hybrid polymer with enhanced emulsifying activity and foaming properties. In fact, the conjugation of whey protein isolate to durian seed gum through Maillard reaction facilitated its migration and absorption at the oil-water interface, thus enhancing its interface activity. The current study revealed that the presence of the protein in the molecular structure of the conjugated polymer significantly affected its foaming ability by reducing the surface tension. In fact, the conjugated polymer showed more hydrophobicity characteristics, better foaming capacity and more efficient emulsifying activity than the non-conjugated durian seed gum. In this study, the non-conjugated and conjugated durian seed gum showed more similar functional properties to Arabic gum rather than the other studied gums (*i.e.*, sodium alginate, carrageenan and pectin). In the present work, Arabic gum and the conjugated durian seed gum exhibited the weakest water holding capacity and widest specific surface among all samples. They also provided the most desirable particle uniformity, foaming capacity and stability among all studied gums. The present work recommends optimizing the conjugation process of durian seed gum and comparing other modification treatments (such as microwave) for future study.
